# Supplementary material for: Investigating the influence of physiologically relevant hydrostatic pressure on CHO cell batch culture
Source: Sci Rep. 2021 Jan 8;11:162. doi: 10.1038/s41598-020-80576-8 (PMC7794228; doi:10.1038/s41598-020-80576-8)
Supplement: Supplementary file 1 — Supplementary Information 1. [file 41598_2020_80576_MOESM1_ESM.zip › S1/IgG/Feb14 0 30 60 90/90mmHg Feb8 2.pdf]

Data File C:\Users\P...hemStation\1\Data\TK_CHO_IgG 2019-02-14 14-17-55\040-9-ML 90 Feb8.D
Sample Name: ML 90 Feb8

===

Acq. Operator : SYSTEM   Seq. Line : 40
Sample Operator : SYSTEM
Acq. Instrument : HPLC-0039   Location : 9
Injection Date : 2/14/2019 5:06:35 PM   Inj : 1
Inj Volume : 100.000 µl
Method : C:\Users\Public\Documents\ChemStation\1\Data\TK_CHO_IgG 2019-02-14 14-17-55\CHO_IgG_LC.M (Sequence Method)
Last changed : 2/8/2019 3:17:23 PM by SYSTEM

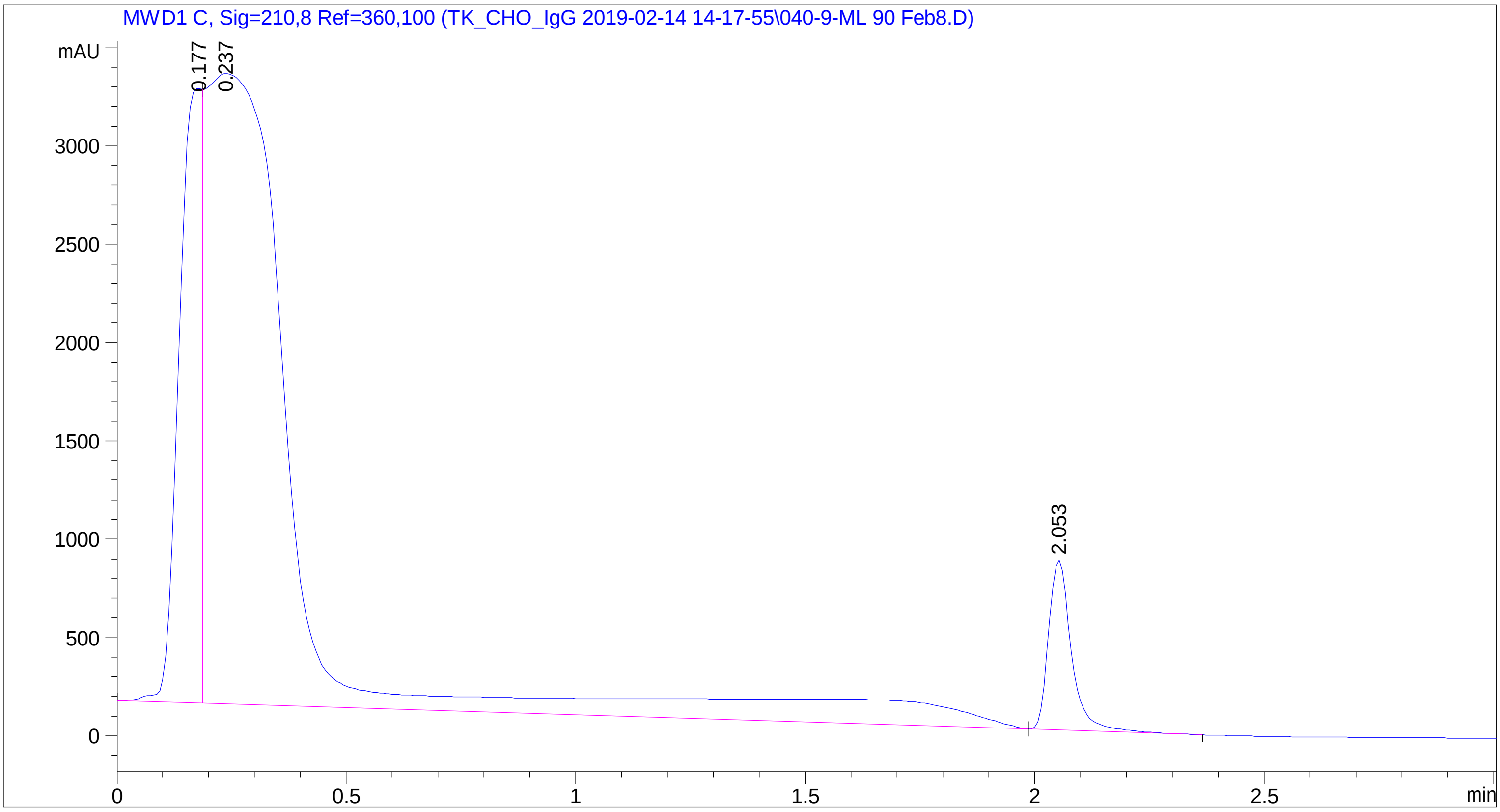

===

Area Percent Report

===

Sorted By : Signal
Multiplier : 1.0000
Dilution : 1.0000
Use Multiplier & Dilution Factor with ISTDs

Signal 1: MWD1 C, Sig=210,8 Ref=360,100

Peak #	RetTime [min]	Type	Width [min]	Area [mAU*s]	Height [mAU]	Area %
1	0.177	BV	0.0558	1.06233e4	3125.16943	18.7964
2	0.237	VB	0.1982	4.28950e4	3204.24219	75.8964
3	2.053	BBA	0.0526	2999.53833	862.20392	5.3072

Totals : 5.65179e4 7191.61554

===

*** End of Report ***
